# Exacerbation of Atopic Dermatitis Associated with Ustekinumab Treatment in Crohn's Disease

**DOI:** 10.7759/cureus.29718

**Published:** 2022-09-28

**Authors:** Brendan Andres, Sasha Taleban

**Affiliations:** 1 Internal Medicine, University of Arizona College of Medicine - Tucson, Tucson, USA; 2 Gastroenterology and Hepatology, University of Arizona College of Medicine - Tucson, Tucson, USA

**Keywords:** t-helper immune response, atopy, ustekinumab, crohn's disease, atopic dermatitis

## Abstract

Ustekinumab inhibits interleukins 12 and 23 and modulates the T helper cell-mediated immune response of Crohn's disease. However, ustekinumab may also exacerbate atopic disease by increasing the T helper 2 cell-mediated pathway. We present the first known case of exacerbation of atopic dermatitis in a patient with Crohn's disease receiving ustekinumab. Additional associations in dose frequency, peripheral eosinophilia, and elevated serum IgE were observed. However, while novel in Crohn's disease, exacerbation of atopy after ustekinumab infusion has been observed in patients with psoriasis and psoriatic arthritis.

## Introduction

Originally used for the treatment of psoriasis and psoriatic arthritis, ustekinumab is approved for moderate to severe Crohn's disease. The T helper (Th) cells, Th-1 and Th-17, play a significant role in the pathogenesis of Crohn's disease. Ustekinumab binds the p40 subunit of interleukin (IL)-12 and IL-23, modulating the lymphocyte response by shifting towards the Th2 immune response due to inhibition of interferon-γ (IFN-γ​​​​​​​). The Th2 pathway may exacerbate atopic diseases, including asthma, atopic dermatitis, and seasonal allergies/hay fever. However, there have been no published cases of atopic dermatitis exacerbation in patients with inflammatory bowel disease receiving ustekinumab.

This article was previously presented as a poster presentation at the 2021 American College of Gastroenterology Annual Scientific Meeting.

## Case presentation

We report a case of a 27-year-old female with a history of atopic dermatitis, seasonal allergies, hay fever, and stricturing upper gastrointestinal and small bowel Crohn's disease. In 2004, she developed diarrhea and failure to thrive. A diagnosis of Crohn's disease was made. She was started on prednisone and azathioprine but did not have a clinical response. In 2004, she was subsequently started on first infliximab and then adalimumab, both of which led to serum sickness-like reactions. At that point, she was continued on azathioprine monotherapy and developed a terminal ileal stricture requiring resection in 2011. In 2011, she was started on certolizumab and methotrexate, which she continued until 2014 when they were stopped after she developed a psoriasiform rash and oral ulcers. She was restarted on azathioprine. In 2015, she began vedolizumab infusions. She had a primary nonresponse and was started on ustekinumab along with azathioprine in September 2015. In March 2016, she retained a capsule endoscopy and was noted to have a mid-ileal stricture that was resected. After her operation in 2016 and in combination with azathioprine, her ustekinumab frequency was increased from every eight to every six weeks. In October 2016, her colonoscopy revealed Rutgeert's i0 disease. She was continued on every six-week ustekinumab injections and azathioprine.

**Figure 1:**
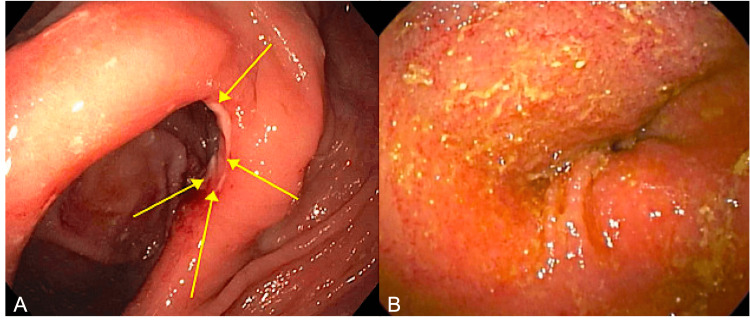
Endoscopic evidence of active ileo-colonic Crohn's disease observed via lower device-assisted enteroscopy A) Rutgeert's i2 ulceration of the end-to-side ileo-colonic anastomosis. B) Erythema of the distal ileum.

In March 2018, she had Rutgeert's i2 disease and erythema of the distal ileum demonstrated during lower device-assisted enteroscopy (Figure 1). In the spring of 2019, the ustekinumab frequency was increased from every six to every four weeks for six months. Subsequently, the patient developed pruritic, eczematous plaques located bilaterally on her dorsal hands, antecubital fossae, and popliteal fossae, all associated with diffuse xerosis. The rash worsened particularly right after her ustekinumab injections and was unresponsive to topical steroid therapy, including clobetasol cream 0.05% twice daily. Previously, since her atopic dermatitis diagnosis, similar rashes had been controlled with topical clobetasol and fexofenadine. Allergy and dermatology were consulted. Subsequent laboratory findings were significant for peripheral eosinophilia, 1.90 K/ul (reference range: 0.00-0.70 K/ul), and elevated serum IgE (sIgE) of 3873 IU/mL (reference range** **≤ 100 IU/mL). Given the lesion morphology, lesion distribution, her history of chronic relapsing atopic dermatitis, and atopy in the context of diffuse xerosis, she was diagnosed with an exacerbation of atopic dermatitis by both allergy and dermatology. Triamcinolone, pimecrolimus, and hydroxyzine were additionally prescribed, but her symptoms remained refractory. No longer controlled with topical agents, there was concern the patient would require additional biologic therapy for atopic dermatitis. Due to concern that IL-12/IL-23 inhibition may have contributed to the worsening of her atopic dermatitis, her ustekinumab frequency was lengthened from every four to every eight weeks. Thereafter, the patient's atopic dermatitis remained controlled with topical therapy. The peripheral eosinophilia resolved; however, sIgE was not re-evaluated. Her Crohn's disease is in clinical and endoscopic remission on ustekinumab infusions every eight weeks and azathioprine.

## Discussion

This is the first reported case of exacerbation of underlying atopic dermatitis associated with ustekinumab in a patient with inflammatory bowel disease. The patient's worsening atopic dermatitis with ustekinumab appears dose-dependent and associated with eosinophilia and elevated sIgE.

The phase III and phase IV studies of ustekinumab in patients with Crohn's disease do not report exacerbation of atopic diseases as an adverse event [1-3]. Similarly, the pooled safety data from the phase II and III trials in patients with psoriasis and psoriatic arthritis did not show that ustekinumab significantly increased exacerbations of atopic diseases [4]. However, several case reports of psoriatic patients developing atopic dermatitis after ustekinumab injection initiation have been published. The authors reported an exacerbation of atopic dermatitis in a patient with a history of childhood atopy. After a period of remission, the patient resumed ustekinumab with a resolution of psoriasis though the patient subsequently experienced an exacerbation of atopic dermatitis that persisted through twelve months of follow-up after cessation [5]. Ustekinumab was further linked to two cases of paradoxical onset atopic dermatitis in patients with psoriasis, one of whom had a history of atopic dermatitis. After initiation of ustekinumab, both patients experienced complete remission of psoriatic skin lesions. However, both proceeded to develop atopic dermatitis that improved rapidly upon ustekinumab cessation [6]. All three of the aforementioned patients experienced eosinophilia and elevated sIgE.

The pathogenesis of atopic dermatitis is complex and multifactorial, with etiologies comprised of genetic, epigenetic, mechanical, immunologic, and bacterial variables. The development of atopic dermatitis in the setting of ustekinumab may be secondary to a more robust Th2 immunogenic response likely exacerbated by ustekinumab's inhibition of INF**-**γ. The effect increases inflammation through IgE production, eosinophil recruitment, and decreased skin barrier integrity from altered free fatty acid, ceramide, and serine protease compositions. Moreover, genetic and epigenetic differences in the production of filaggrin, a protein important in skin barrier integrity, can impact a patient's susceptibility to atopic dermatitis [7]. Additionally, atopic dermatitis and Crohn's disease are linked through dysbiosis in the gut microbiome that leads to decreased production of anti-inflammatory butyrate and propionate. Furthermore, the "leaky gut" phenomenon occurring in Crohn's disease is hypothesized to augment the Th2 response within the skin via the passage of novel pathogens through the gut epithelium [8]. The gut microbiome is further altered after ustekinumab-induced remission of Crohn's disease [9]. 

The pharmacokinetics of ustekinumab are dose- and frequency-dependent. Ustekinumab achieves a steady state after the second maintenance dose and has a half-life of 14.9 to 45.6 days. Mucosal inflammation has been suggested to mediate ustekinumab pharmacokinetics as well. Sandborn et al. and Feagan et al. demonstrated trough levels of 2.1 and 6.4 μg/mL, respectively, for the 130 mg and 6 mg/kg dose groups eight weeks after induction [1,2]. Adedokun et al. observed a three-fold increase in trough levels between eight and twelve-week frequencies, 2.11 vs. 0.62 mg/mL, respectively [10]. Attempts to demonstrate cutoff values for endoscopic remission for ustekinumab in Crohn's disease patients have further illustrated that serum concentrations increase with increased dose frequency. In patients with small bowel Crohn's disease, Hirayama et al. demonstrated mean ustekinumab trough levels of 3.3 and 1.8 μg/mL, respectively, for patients with and without endoscopic remission while on maintenance ustekinumab 90 mg every eight weeks [11]. In patients with Crohn's disease who were refractory or intolerant to anti-TNF therapy, Battat et al. observed mean ustekinumab trough concentrations of 4.7 and 3.8 μg/mL in patients with and without endoscopic response, respectively, while on maintenance therapy of ustekinumab 90 mg primarily every four weeks (42 of 56 patients) [12]. Finally, increased mucosal inflammation has been proposed to decrease ustekinumab trough levels by increased production of IL-12 and IL-23, which then consumes ustekinumab [11]. Numerous studies which exhibit that intensifying ustekinumab decreases loss of response in Crohn's disease patients support this mechanism [13-17].

In our patient, we hypothesize that a history of atopy increased susceptibility to a Th2 response. After the resolution of her Crohn's disease flare, ustekinumab augmented the Th2 response due to increased serum concentration from more frequent dosing and decreased consumption from mucosal inflammation. Her atopic dermatitis subsequently improved after decreasing the frequency of ustekinumab, likely due to lowered serum concentrations.

## Conclusions

In conclusion, ustekinumab may exacerbate atopic dermatitis in patients with Crohn's disease and underlying atopy due to a suspected shift in the Th immune response. Exacerbation is likely dose-dependent and associated with serologic markers of atopy. Further studies are needed to confirm an association between ustekinumab and atopic dermatitis in patients with Crohn's disease. Confirmation could provide additional aid in selecting biologic therapy for patients with Crohn's disease.
